# Nucleotide Sequence Sharing between the Human Genome and Primers for Severe Acute Respiratory Syndrome Coronavirus 2 (SARS-CoV-2) Detection

**DOI:** 10.1055/s-0042-1743260

**Published:** 2022-06-13

**Authors:** Darja Kanduc

**Affiliations:** 1 Department of Biosciences, Biotechnologies, and Biopharmaceutics, University of Bari, Bari, Italy

**Keywords:** PCR primers, SARS-CoV-2 detection, false positives

## Abstract

This study shows that oligonucleotide sequences are shared between the human genome and primers that have been proposed/used for SARS-CoV-2 detection by polymerase chain reaction (PCR). The high level of sharing (namely, up to 19mer with a maximum number of gaps equal to 2) might bear implications for the diagnostic validity of SARS-CoV-2 detection by PCR.

## Introduction

Defining the relationship(s) between infectious agents and the human host is a crucial topic in immunology, microbiology, and infectious medicine. Although it has been proposed that genetic factors might play a role, 1 2 the exact mechanisms of chronic infections and occasional (re)activation of pathogens in the human host are largely misunderstood and poorly studied. The issue became even more relevant in light of the recent Ebola virus, Dengue virus, and SARS outbreaks associated with high morbidity and mortality. 3 4 5 In this context, there is a need not only for knowing the molecular basis of infections to define effective and safe preventive and therapeutic interventions but also for sensitive and specific diagnostic tools. Indeed, accurate screening of asymptomatic, presymptomatic, and symptomatic subjects might be key to effective epidemiological measures during pandemics. However, especially in analyzing SARS-CoV-2 as a paradigmatic example, contrasting data have been reported on the analytical performance of SARS-CoV-2 detection methods and claims about the rates of false negatives and false positives have been published. 6 7 8 9 10 11

On the basis of all these, this study focused on the possible genetic basis of potential false polymerase chain reaction (PCR) results by comparing the nucleotide sequence of proposed/used SARS-CoV-2 primers versus the human genome. The scientific rationale is that—given the high level of amino acid sequence sharing between SARS-CoV-2 proteins and the human proteome 12 13 14 15 —parallel sequence matching at the nucleotide level might exist between the SARS-CoV-2 primer sequences and the human genome, in this way possibly explaining the generation of false-positive SARS-CoV-2 detection results. Data are reported here that confirm the likelihood of the research hypothesis.

*De facto* , using the nucleotide Basic Local Alignment Search Tool (BLASTn) program from NCBI ( http://blast.ncbi.nlm.nih.gov , 16 17 a sample of 12 primers retrieved from literature, 18 19 proposed/used even by government health institutions 19 to detect SARS-CoV-2, and described here in Table 1 , was analyzed for nucleotide sequence sharing with the human genome. BLASTn analyses documented a relevant viral versus human oligonucleotide overlap, with shared primer sequences repeatedly present in the human genome, disseminated among different chromosomes, and located in plus strands, minus strands, mRNAs, pseudogenes, etc. Due to space constraints, an *in extenso* description of the complete nucleotide sequence sharing is practically not possible, and only a synthetic snapshot is shown in Table 2 .

**Table 1:** Nucleotide sequence of primers used/proposed for PCR detection of SARS-CoV-2 a

Primer no.	Target gene b	Primer direction	Primer nucleotide sequence
1	S 2	F	CCACTAGTCTCTAGTCAGTGTGTTAAT
2	S 2	R	AAACTGAGGATCTGAAAACTTTGTC
3	8	F	GGAGCTAGAAAATCAGCACCTTTAA
4	8	R	TCGATGTACTGAATGGGTGATTTAG
5	E	F	ACAGGTACGTTAATAGTTAATAGCGT
6	E	R	ATATTGCAGCAGTACGCACAGA
7	N	F	GACCCCAAAATCAGCGAAAT
8	N	R	TCTGGTTACTGCCAGTTGAATCTG
9	N	F	GGGGAACTTCTCCTGCTAGAAT
10	N	R	CAGACATTTTGCTCTCAAGCTG
11	N	R	TAATCAGACAAGGAACTGATTA
12	N	F	TGGCAGCTGTGTAGGTCAAC

Abbreviations: F, forward; PCR, polymerase chain reaction; R, reverse.

a Primers retrieved from Gadkar et al 18 and Qasem et al, 19 and further details and references therein.

b Gene names given according to Uniprot. 20

**Table 2:** Oligonucleotide sharing between the human genome and PCR primers proposed/used to detect SARS-CoV-2: a few examples a

a Twelve primers described in Table 1 and derived from [ 18 19 ] were analyzed for sharing of nucleotide sequences with the human genome. BLASTn [ 16 17 ] was used to find and localize regions of identity in the human nucleotide collection covering genomic and transcript sequences; further details are available at http://blast.ncbi.nlm.nih.gov . The 12 primers are listed with shared nucleotide sequences given underlined.

In conclusion, this communication highlights the likelihood that viral versus human nucleotide sequence overlap can interfere with nucleic acid amplification testing and generate PCR false-positive results in SARS-CoV-2 detection, in this way affecting medical diagnoses.
